# Amber suppression coupled with inducible surface display identifies cells with high recombinant protein productivity

**DOI:** 10.1002/bit.26892

**Published:** 2019-01-18

**Authors:** Lina Chakrabarti, Li Zhuang, Gargi Roy, Michael A. Bowen, William F. Dall’Acqua, Pam Hawley‐Nelson, Marcello Marelli

**Affiliations:** ^1^ Cell Culture and Fermentation Science, MedImmune Gaithersburg Maryland; ^2^ Antibody Discovery and Protein Engineering, MedImmune Gaithersburg Maryland

**Keywords:** amber suppression, cell line development, enrichment, surface display, unnatural amino acids

## Abstract

Cell line development (CLD) for biotherapeutics is a time‐ and resource‐intensive process requiring the isolation and screening of large numbers of clones to identify high producers. Novel methods aimed at enhancing cell line screening efficiency using markers predictive of productivity early in the CLD process are needed to reliably generate high‐yielding cell lines. To enable efficient and selective isolation of antibody expressing Chinese hamster ovary cells by fluorescence‐activated cell sorting, we developed a strategy for the expression of antibodies containing a switchable membrane‐associated domain to anchor an antibody to the membrane of the expressing cell. The switchable nature of the membrane domain is governed by the function of an orthogonal aminoacyl transfer RNA synthetase/tRNApyl pair, which directs a nonnatural amino acid (nnAA) to an amber codon encoded between the antibody and the membrane anchor. The process is “switchable” in response to nnAA in the medium, enabling a rapid transition between the surface display and secretion. We demonstrate that the level of cell surface display correlates with productivity and provides a method for enriching phenotypically stable high‐producer cells. The strategy provides a means for selecting high‐producing cells with potential applications to multiple biotherapeutic protein formats.

## 1 INTRODUCTION

Antibodies and antibody‐derived molecules, representing the best‐selling class of biotherapeutics are efficiently produced and secreted by mammalian cells, with Chinese hamster ovary (CHO) cells being the major host used for manufacture (Walsh, 2010). Although improvements in expression levels have been achieved through vector engineering and cell culture process optimization, the identification and selection of high‐yielding stable cell lines are labor‐, time‐, and resource‐intensive activities (Wurm, 2004; Zhu & Hatton, 2017). Typically, hundreds of transfectants are grown from single cells in static microtiter plates and assessed for productivity in multiple sequential screens to identify high‐titer clones. Early stage screening steps often show limited predictability of later stage fed‐batch culture productivity, where medium, feed, and culture conditions influence growth, viability, and ultimately titers (Handlogten, Zhu, & Ahuja, 2018; Roy, Zhang et al., 2017). Advances in cell line development (CLD) technologies that improve the early detection and identification of high‐producing cells are therefore crucial to generate high‐producing cell lines and supporting the rapid development of biotherapeutic antibodies. Approaches have been developed to detect monoclonal antibody (mAb) on the cell surface, using covalent or noncovalent anchoring strategies (Helman et al., 2014; Kumar & Borth, 2012; S. Lang et al., 2016). Fluorescence‐activated cell sorting (FACS) and magnetic bead selection processes then allow high‐throughput identification and enrichment of producing cells based on immobilization of the desired secreted proteins on the cell surface (Pichler et al., 2009). Methods including cold‐capture (Brezinsky et al., 2003), matrix‐aided surface capture (Böhm et al., 2004; Holmes & Al‐Rubeai, 1999), and gel microdrop technology (Hammill, Welles, & Carson, 2000; Powell & Weaver, 1990) have successfully shown enrichment of productive cells after several rounds of selection. However, relatively modest expression levels (<1 g/L) have been reported, raising the question of whether these methods can discriminate cells with higher expression levels. Other FACS‐based enrichment technologies include the use of reporter proteins, such as GFP (Meng, Liang, Wong, & Chisholm, 2000), or nonfluorescent reporter molecules directed to the cell surface, such as CD4, CD20, or CD52 that are cotranslated with the target antibody using an IRES element (Bailey, Tait, & Sunstrom, 2002; Cairns et al., 2011; DeMaria et al., 2007; Helman et al., 2014). These methods have shown good correlation between reporter expression and mAb productivity and proven useful for the enrichment of high‐expressing cells. However, here too, modest expression levels are described, and the constitutive expression of reporter proteins may generate undesirable impurities in biopharmaceutical manufacturing, potentially complicating purification, and product analysis.

In an effort to develop a robust and facile method for the selection of cells with very high expression levels, we exploited an amber suppression technology to control the expression of membrane‐anchored antibody, which can be detected on live cells by flow cytometry. The switchable nature of the technology derives from engineering the cells with the ability to incorporate nonnatural amino acids (nnAA) into proteins (Wang et al., 2010). This has been most effectively achieved by reassigning an amber stop codon to a nnAA through the function of an orthogonal transfer RNA (tRNA) synthetase (pylRS) and its cognate tRNA (tRNApyl), derived from the archaebacteria *Methanosarcina mazei* (Mukai et al., 2008; Wan, Tharp, & Liu, 2014). The most common application of this technology is in the introduction of nnAAs containing functional groups that enable biorthogonal conjugations (K. Lang et al., 2012; Nguyen et al., 2009; VanBrunt et al., 2015). In this study, we expand the utility of nnAA incorporation, not through the functionality of the nnAA, but by exploiting the regulation of amber codon readthrough. The specificity of the pylRS/tRNApyl pair for a nnAA ensures that amber suppression activity occurs only when cells are exposed to nnAA; making this process “switchable” in response to the addition, or removal, of the nnAA in the medium. Thus, by encoding an amber stop codon at the C‐terminus of the gene of interest, followed by an in‐frame glycosylphosphatidylinositol‐membrane anchoring domain (GPI anchor), we can regulate the expression of the fusion and its subsequent display on the membrane of the expressing cell. The displayed antibody represents a product‐related reporter for the productivity of the cells and can be readily detected in live cells using FACS. Similar methods exploiting amber suppression using alternative splicing (Horlick et al., 2013), leaky stop codons (S. Lang et al., 2016), chemically induced suppression (Bouquin, Rasmussen, Bertilsson, & Okkels, 2006), or using protease activity (Chuang et al., 2014), have been described for the same purpose and have demonstrated their utility for the selection of expressor cells. However, the extended exposures to antibiotics, inhibitors, and perpetual expression of reporters required to operate these technologies limit the utility and range of expression levels that can be discriminated. Using CHO cells capable of nnAA incorporation, we demonstrate that the levels of antibody surface display closely correlates with the fed‐batch productivity of the cells. We provide evidence to support the use of this technology to enrich for stable high‐producer cells by FACS, early in the cell line engineering process, to generate high‐producing pools and clonal cell lines. Finally, we demonstrate the utility of this system for selectively enriching the expressor cells for very low expressing complex therapeutic proteins.

## 2 MATERIALS AND METHODS

### 2.1 Vector construction

**Figure 1:**
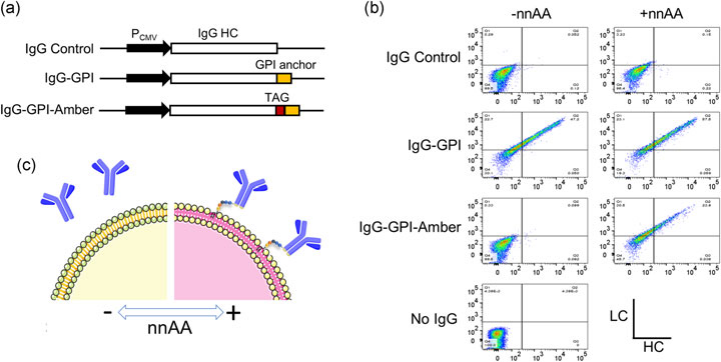
Orthogonal pylRS/tRNA pair enables switchable IgG membrane display. (a) Constructs for the expression of an antibody heavy chain either alone (IgG control), fused to a glycosylphosphatidylinositol (GPI) membrane anchor (IgG‐GPI) or IgG‐GPI containing an in‐frame amber stop codon (TAG) between the IgG HC and GPI anchor (IgG‐GPI‐Amber). (b) CHO‐RS cells capable of efficient amber suppression in the presence of an nnAA were transiently transfected with IgG control, IgG‐GPI and IgG‐GPI‐Amber constructs and stained for membrane‐bound IgG. Cells expressing the IgG‐GPI showed surface staining in cells in the presence and absence of nnAA whereas IgG‐Control cells showed low surface staining in both conditions. IgG‐GPI‐Amber showed surface staining in the presence of nnAA, but not in its absence. No‐expression IgG (No IgG) cells were used as negative controls for surface staining in the absence of IgG expression. (c) In cells expressing the pylRS/tRNA pair, nnAA enables efficient amber codon readthrough and expression of the membrane anchor fusion. In the absence of nnAA, the pylRS/tRNA pair is silent resulting in the termination of translation at the amber codon and expression of a secreted antibody. CHO: Chinese hamster ovary; HC: heavy chain; IgG: immunoglobulin G; nnAA: nonnatural amino acids; tRNA: transfer RNA [Color figure can be viewed at wileyonlinelibrary.com]

Genes encoding a heavy chain (HC) and light chain (LC) of an immunoglobulin G (IgG) directed to ephA2 were placed under control of cytomegalovirus (CMV) promoters near expression‐enhancing elements in the plasmid vector pCLD (IgG control). A membrane‐associated IgG was made by expressing the HC fused to the glycosylphosphatidylinositol‐membrane anchor sequence (IgG‐GPI). An amber codon was inserted in frame at the HC‐GPI junction to generate a reversible membrane‐anchored IgG (IgG‐GPI‐Amber; Figure 1a). All antibody vectors encode glutathione synthetase (GS) under control of the SV40 promoter that allows for the selection of cells in methionine sulfoximine (MSX; Bebbington et al., 1992). pCLD‐puro‐pylRS‐tRNA is a proprietary plasmid containing a puromycin resistance marker under control of the SV40 promoter, a CMV‐pylRS expression cassette, and 18 tandem repeats of the tRNApyl gene under the control of the U6 small nuclear RNA promoter. pRFP‐GFPamb is a reporter plasmid construct encoding an mCherry red fluorescent protein (RFP)–green fluorescent protein (GFP) fusion containing an amber codon between the RFP and GFP fluorophores.

### 2.2 Cell culture and transfection

Methods for the generation of host cells capable of nnAA incorporation have been previously described (VanBrunt et al., 2015). In brief, a proprietary cell line derived from CHO‐K1 was adapted to suspension cell growth and selected for low levels of glutamine synthetase expression. This cell line was used as the host for the pylRS/tRNA system (Roy, Martin et al., 2018; Roy, Zhang et al., 2017). The engineered cells were generated by transfection with the pCLD‐puro‐pylRS‐tRNA plasmid and selected in medium containing 6.5 µg/ml puromycin. The wild‐type pylRS has been shown to recognize a variety of nnAAs and in this study, we utilized N6‐([2‐azidoethoxy]carbonyl)‐l‐lysine (Synchem, Chicago, IL) as the nnAA for amber suppression (Wan et al., 2014). Stable transfectant isolates were expanded and transiently transfected with the pRFP‐GFPamb plasmid and grown in the presence of 2 mM nnAA for 16–24 hr. Isolates showing the best RFP:GFP ratios were further characterized for their ability to incorporate nnAA into a target IgG at a high titer. The best cell line (CHO‐RS) was selected for the evaluation of surface display. CHO‐RS was maintained in CD‐CHO medium containing puromycin. Stable antibody expressors were generated in CHO‐RS by nucleofection of linearized IgG expression plasmids. Transfected cells were allowed to recover in CD‐CHO medium (Thermo Fisher Scientific, Frederick, MD) for 48 hr, followed by selection in CD‐CHO containing 50 µM MSX and 6.5 µg/ml puromycin. Stable transfectants were maintained in CD‐CHO containing MSX and puromycin. Transient transfections were also conducted in CHO‐RS using nucleofection and following the manufacturer’s recommendations (Lonza, Walkersville, NC). Following transfection with the IgG expression plasmids, cells were grown in CD‐CHO containing 2 mM nnAA for 16 hr before labeling and flow cytometric analyses.

### 2.3 Flow cytometry analysis for surface display

Ten million transfected cells expressing membrane‐anchored IgG were centrifuged at 300*g* for 5 min, resuspended in a 10 ml fresh culture medium containing nnAA and incubated for 2 or 4 hr shaking at 120 rpm at 37°C. Following nnAA treatment, 1 × 10^6^ cells were centrifuged at 300*g* for 5 min, washed with FACS buffer (1% fetal bovine serum in 1× phosphate‐buffered saline [PBS]) and stained with goat anti‐human IgG (Fc)‐Alexa Fluor 488 (Life Technologies, Carlsbad, CA) and goat anti‐human kappa‐APC (Biolegend, San Diego, CA) for 15 min at room temperature. The surface stained cells were washed and resuspended in FACS buffer for flow cytometry analysis in an LSRII instrument (BD Biosciences, San Jose, CA). Data analysis was performed using FlowJo software (Tree Star, Inc., Ashland, OR).

### 2.4 Intracellular staining for antibody expression level

Intracellular expression of the heavy and light chains of antibody molecules was determined by staining cells with fluorescently‐labeled antibodies specific for heavy or light chains (Roy, Miro‐Quesada, et al., 2017). Briefly, the cells were centrifuged, washed with FACS buffer and fixed with Fixation Medium A (Life Technologies) for 15 min at room temperature. Next, the cells were washed with FACS buffer and stained for 15 min at room temperature with the staining solution comprised of goat anti‐human IgG (Fc)‐Alexa Fluor(AF)488 (Life Technologies) and goat anti‐human κ‐APC (Biolegend) in Permeabilization Medium B (Life Technologies). The stained cells were washed and resuspended in FACS buffer before analysis on an LSRII for the APC and AF488 double‐positive population. Data analysis was performed using FlowJo software.

### 2.5 Cell propagation

Cells were seeded at 3 × 10^5^ cells/ml in 30 ml CD‐CHO medium in 125‐ml Erlenmeyer flasks and grown at 37°C, 6% CO_2_, and 120 rpm on an orbital shaking platform. Cells were passaged every 3–4 days following measurement of viable cell density and viability using a ViCell Automated Cell Counter (Beckman Coulter, Brea, CA).

### 2.6 Fed‐batch culture

Antibody production was evaluated by fed‐batch culture in a proprietary medium in 125‐ml Erlenmeyer flasks or 96 deep wells in the absence of puromycin. The production cultures were grown at 35.5°C in a humidified 6% CO_2_ atmosphere for up to 13 days unless otherwise mentioned. Shaker speed was maintained at 120 rpm for flasks and 350 rpm for 96 deep wells (Roy, Martin, et al., 2018). Cell density and viability were monitored during cultivation in flasks. Proprietary feed was added to the production cultures on alternate days starting on Day 3. Antibody titers in culture supernatant were determined using Protein A biosensors in an Octet QK384 (Pall ForteBio, Fremont, CA).

### 2.7 Cell sorting

Bulk cell sorting and single‐cell deposition cloning was performed using a BD Influx cell sorter (BD Biosciences) based on a method described previously (Evans et al., 2015). For the bulk sort, 2 × 10^7^ cells were treated with nnAA for 2 or 4 hr and harvested by centrifugation. The cells were washed and stained with AF488‐conjugated anti‐human IgG (Fc specific) using sorting buffer containing PBS, 0.5% recombinant human serum albumin (Sigma, St. Louis, MO), 5 mM ethylenediaminetetraacetic acid (Life Technologies) and 25 mM HEPES (Calbiochem, San Diego, CA). Based on high and low AF488‐fluorescence intensity gated fractions, 2.5 × 10^5^ cells were deposited into 5‐ml collection tubes containing the culture medium. The sorted cells were centrifuged, resuspended in 2.5 ml fresh culture medium and plated into six‐well plates. For single‐cell cloning, 1 × 10^6^ cells (nnAA‐treated and stained with AF488‐conjugated anti‐human IgG [Fc specific] antibody) were sorted from the AF488‐gated fraction by depositing one cell per well into individual wells of 384‐well plates containing conditioned medium. All plates were incubated at 37°C in a humidified atmosphere with 6% CO_2_ for outgrowth.

### 2.8 Statistical analysis

One‐way analysis of variance (ANOVA) was used to determine whether the differences in the means of expression groups were statistically significant with a 95% confidence interval. The Dunnett test was performed to compare the high surface‐display group to each of the other groups within GraphPad Prism 7.04 Software (San Diego, CA).

### 2.9 Intact mass analysis

For MS analysis, a sample containing 2–5 mg of protein was reduced with 10 mM dithiothreitol at 37°C for 10 min. Samples were then desalted on a protein macrotrap column (Optimize Technologies, Oregon City, OR) by washing with 0.1% formic acid in water, then eluted with 70% acetonitrile, 0.1% formic acid into an Agilent 6520 Q‐TOF MS (Santa Clara, CA). Deconvolution was performed using Agilent Bio‐confirm Software (Santa Clara, CA).

## 3 RESULTS

### 3.1 Evaluation of surface‐display platform cells

To establish whether a switchable IgG membrane anchor could be effectively displayed and visualized on the cell surface of CHO cells, CHO‐RS was transiently transfected with expression plasmids encoding a well‐expressed human IgG1 directed against the tumor antigen EphA2 (Jackson et al., 2008; Peng, Oganesyan, Damschroder, Wu, & Dall’Acqua, 2011). Three different IgG expression vectors were generated in which the HC gene was expressed: (a) without a fusion partner to generate an IgG control, (b) fused to a GPI anchor, or (c) fused to a GPI anchor that also contains an amber stop codon before, and in frame with, the GPI cassette (IgG‐GPI‐Amber; Figure 1a). Each construct was transiently transfected into CHO‐RS and grown for 12 hr in the presence, or absence, of nnAA. To assess whether discrete membrane staining was observable, the cells were immunostained for membrane‐bound LC and HC and analyzed by flow cytometry (Figure 1b). As expected, low, but detectable, levels of the surface display were observed in cells expressing the antibody lacking the GPI anchor (IgG control). In cells transfected with IgG‐GPI, greater than 50% of the population showed LC^+^HC^+^ staining (IgG‐GPI) in both the presence as well as the absence of nnAA. In contrast, cells expressing the IgG‐GPI‐Amber construct showed 24% double positive cells in the presence of nnAA, but greatly reduced cell surface staining in the absence of nnAA similar to the IgG control samples, indicating no significant accumulation of IgG on the cell surface. These results show a distinguishable amber suppression‐dependent accumulation of IgG‐GPI at the cell surface in response to nnAA for the IgG‐GPI‐Amber construct (Figure 1c).

To determine whether this display was applicable to stable pools, surface binding was examined in cells stably transfected with the IgG‐GPI‐Amber construct in a vector containing expression‐enhancing elements that have been shown to decrease transgene silencing and provide consistent high‐level expression of target proteins. Thus, CHO‐RS was transfected with pCLD‐IgG‐GPI‐Amber and selected in medium containing MSX. Transfectant pools were expanded and grown in the presence of nnAA to induce the surface display of the IgG. To identify induction conditions that allowed for discrimination of expressors various concentrations of nnAA and times of treatment were examined (Supporting Information Figure S1). In these cells, we showed the advent of surface display with very low concentrations of nnAA and at very early time points (2–4 hr). The levels of surface staining increased with increased nnAA concentration and longer incubation time. Based on these data, cells were incubated in 25 µM nnAA for 2 hr for FACS selection, to provide a diversity of cell surface staining while maintaining Subsaturating levels of the membrane IgG.

The switchable nature of the surface display is achieved in a host cell line engineered to express pylRS and tRNApyl which directs nnAAs to amber codons. To maintain consistent amber suppression efficiency across a population, cell lines stably expressing these genes were generated and functionally selected for high amber suppression efficiency (VanBrunt et al., 2015). Thus, the expression of these genes may result in global amber codon readthrough resulting in the extension of essential proteins (25% of proteins are terminated by amber codons) that may affect the cell's growth and viability. This is a significant concern for highly efficient amber‐suppressor cells when exposed to nnAA, however, the specificity of the pylRS for nnAA makes this unlikely in cells grown in the absence of nnAA (VanBrunt et al., 2015). Nevertheless, the burden of expressing pylRS and tRNApyl on cell growth and viability was examined in CHO‐RS (Supporting Information Figure S2). CHO‐RS and its parental cell line were grown in shake flasks and cell density and viability were tracked for 7 days. We observed that CHO‐RS and the parental cell line retained similar growth kinetics and viability over the course of the experiment. Furthermore, both cultures achieved very high cell densities (>1.0 × 10^7^ cells/ml) in this time while retaining high viability (>95%). These data show that the engineered host does not have altered growth properties relative to its parental lineage under these conditions. The technology presented here requires minimal exposure of the cells to nnAA to activate the surface display and thus, deleterious effects on growth were not observed.

### 3.2 Enrichment based on amber suppression–dependent surface display

**Figure 2:**
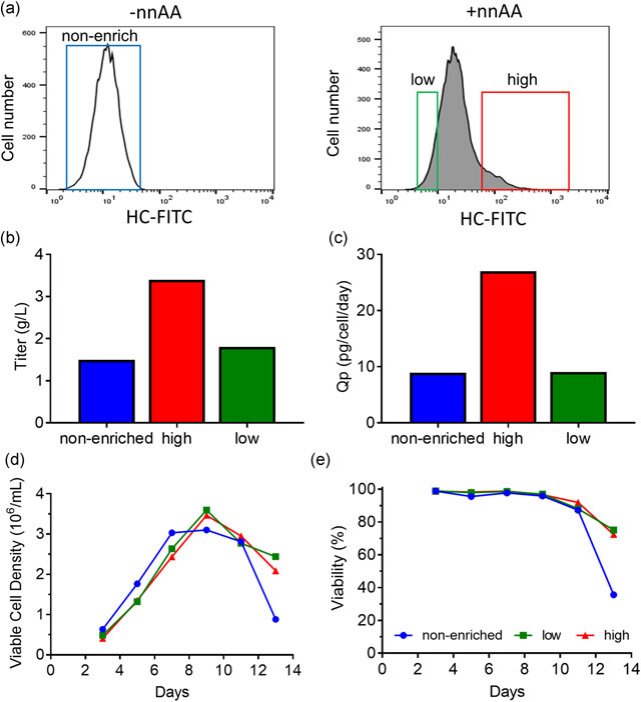
Surface display based selection enhances the expression level of stable pools. (a) Pools of cells stably expressing an IgG‐GPI‐Amber were grown in the absence (−nnAA) or presence of nnAA (+nnAA) and sorted by FACS based on HC expression using the indicated gates to segregate cells into a nonenriched, low and high surface‐display pools. (b, c) Sorted subpools were expanded and their fed‐batch titers and specific productivity (Qp) were determined. Cells sorted from the high surface display showed higher overall titers and Qp than nonenriched or low surface display pools. Viable cell density (d) and viability (e) were determined for all cultures over the course of the 13‐day fed‐batch expression. FACS: fluorescence‐activated cell sorting; FITC: fluorescein isothiocyanate; IgG: immunoglobulin G; GPI: glycosylphosphatidylinositol‐membrane anchoring domain; HC: heavy chain; nnAA: nonnatural amino acid [Color figure can be viewed at wileyonlinelibrary.com]

Having established that we could regulate the display of an antibody on the surface of cells, we then assessed whether the expression levels of membrane‐bound IgG could be used to enrich for high‐producing pools. Stable CHO‐RS pools expressing IgG‐GPI‐Amber were treated with nnAA and sorted into two subpools based on high and low expression levels of membrane‐bound IgG detected by flow cytometry (Figure 2a). In addition, a control population was generated from cells to represent the expression potential of cells without the benefit of surface display based sorting. The cells were derived from a culture that was not activated with nnAA and the entire population gated for sorting (nonenriched). Cultures from the nonenriched, low, and high groups were subcultured and their productivity was measured following 13 days of fed‐batch culture in shake flasks. Cells selected for high surface display showed improved expression levels (3.4 g/L) over cells from the low surface‐display group (1.5 g/L) or nonenriched populations (1.8 g/L; Figure 2b). A similar correlation was observed between the surface display and specific productivity (Qp) of the sorted pools (Figure 2c). All three cultures showed similar cell densities and viability throughout the fed‐batch expression (Figure 2d,e). These data indicate that FACS enrichment for cells with high surface display can be used to improve the productivity of an expression pool.

**Figure 3:**
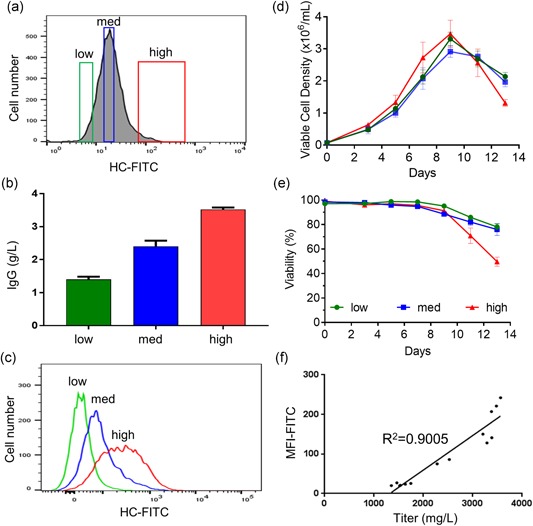
High surface‐display levels correlate with increased titers. (a) Stable IgG‐GPI‐Amber pools were grown in the presence of nnAA and bulk sorted by FACS into low, medium, and high surface‐staining pools. (b) Expression levels of the sorted subpools by fed‐batch cultures were determined in a 13‐day fed‐batch expression (*n* = 3). (c) Surface display of the sorted subpools was measured by flow cytometry. Three overlapping populations with different surface‐staining levels were preserved through the fed‐batch process. (d) Viable cell density and (e) viability of the cultures was monitored throughout the 13‐day fed‐batch expressions. All cultures showed similar growth kinetics indicating that differences in titer are not the result of poor growth. (f) Heavy chain surface display MFI and subpool titers show a strong positive correlation. Coefficient of determination (*R* ^2^) is shown. FACS: fluorescence‐activated cell sorting; IgG: immunoglobulin G; GPI: glycosylphosphatidylinositol‐membrane anchoring domain; MFI: median fluorescence intensity; nnAA: nonnatural amino acid [Color figure can be viewed at wileyonlinelibrary.com]

To further examine whether a correlation exists between productivity and the levels of surface display, a CHO‐RS pool expressing IgG‐GPI‐Amber was sorted into subpools based on low, medium, and high surface display (Figure 3a), postsort cells were expanded for measuring productivity after a 13‐day fed‐batch culture as well as analysis for surface binding. As previously observed, cells from the high (3.5 g/L) surface‐display gate were more productive than cells from either the medium (2.4 g/L) or low (1.4 g/L) surface‐display groups (Figure 3b), and retained differential surface‐staining levels upon retesting (Figure 3c). In all cases, cell culture viability and viable cell densities were comparable, suggesting that differences in expression were not related to cell growth or viability (Figure 3d,e). When titer values were plotted against the median fluorescence intensity (MFI) of membrane‐bound IgG, a correlation coefficient of 0.9005 was calculated indicating that nnAA‐induced amber suppression–dependent surface display can be used as a representative of cell productivity (Figure 3f).

### 3.3 Clonal enrichment of high‐producer cells based on surface display

**Figure 4:**
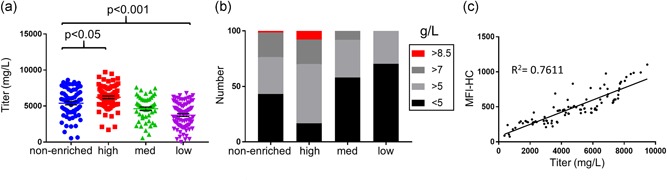
Surface‐display based selection enriches for high expression clones. (a) Single cells expressing the IgG‐GPI‐Amber were sorted from high, medium, and low surface‐display gates (as shown in Figure 3a), and expression levels were measured in 96 deep‐well fed‐batch cultures after Day 13. The expression level of each clone was plotted along with a control population sorted in the absence of surface display. Mean expression values from high surface‐display gates showed a statistically significant difference to medium, low, and nonenriched population. *P* values for the indicated pairs is shown. (b) To highlight the enrichment of high‐producing clones using surface display the percent of clones identified in each group above selected titers were plotted in a stacked graph. High surface‐display selections showed an enrichment in very high‐producing clones (>8.5 g/L; red) than other gates or the nonenriched population. (c) The correlation between the surface display MFI and expression levels was examined in 30 clones from each of the surface‐display gated fractions. A fitted regression line is shown with its coefficient of determination (*R* ^2^). These data show a strong correlation between the surface display and expression titer. IgG: immunoglobulin G; GPI: glycosylphosphatidylinositol‐membrane anchoring domain; MFI: median fluorescence intensity [Color figure can be viewed at wileyonlinelibrary.com]

Generation of clonal manufacturing cell lines is a crucial step towards ensuring reproducible product quality for biopharmaceuticals. FACS is commonly used for cloning antibody‐producing cells (DeMaria et al., 2007), but several studies have reported that multiple rounds of screening and cloning are necessary to ensure the isolation of high producers (Okumura et al., 2015). We sought to determine whether one round of clonal cell sorting based on amber suppression–dependent surface display is adequate to enrich for the highest antibody‐producing cells. Following surface staining of CHO‐RS IgG‐GPI‐Amber stable pools treated with nnAA, single cells were deposited into each well of the 384‐well plates based on low (bottom 5%), medium (mid 5%), and high (top 3%) fluorescence intensity of membrane‐bound IgG HC. A population of control cells was cloned without surface display and staining (nonenriched). A total of 283 clones (high, 77; medium, 50; low, 84; and nonenriched, 72) were screened in 96 deep‐well fed‐batch cultures and analyzed for productivity at Day 13 (Figure 4a). Clones obtained from the top surface‐display gated region showed improved average productivity compared to nonenriched, medium, or low surface‐display derived clones. A one‐way ANOVA of the data showed a statistically significant difference between the means of the high group and each of the other three groups. Importantly, clones selected by the high surface‐display gates showed an enrichment of high expressors (>8.5 g/L) along with a concomitant reduction in low producers (<5 g/L) relative to nonenriched controls (Figure 4b; Supporting Information Table 1). Indeed, our analysis shows that almost 17% of the clones isolated from the high surface‐display group showed productivity greater than 8.5 g/L. This is in contrast to 1.8% of cell lines from the nonenriched group. Furthermore, we saw an underrepresentation of low producers (<5 g/L) in the high surface‐display group (16%) relative to the nonenriched group (43%). This indicates that the selection method greatly increases the identification of high‐producing clones.

Next, we sought to elucidate the correlation between secreted titers and surface‐display membrane intensity in the clonal cells. Thirty‐three clones from each of high, medium, and low surface‐display fractions with various titer levels were selected randomly for this analysis. The individual clonal cells were treated with nnAA, stained for surface binding of LC and HC, and analyzed by flow cytometry. The titers of the clones, when plotted against the MFI of membrane‐bound HC antibody, revealed a positive correlation (*R* ^2^ = 0.7611; Figure 4c). To examine whether the high surface‐display phenotype persisted after expansion, cultures derived from each of the 33 clones in high, medium, and low gates were retested for surface display of the target antibody (Supporting Information Figure S3). The data show that cultures derived from the high gates retained an elevated IgG display and showed a narrow distribution of cells suggesting a high homogeneity within each population. These data indicate that this strategy provides an effective tool for high‐throughput screening for high‐producing cells. Moreover, clones derived from the high surface‐display gate showed the largest numbers of high producers and the top overall titers. The improved identification rate of the highest producing clones allows more options for the selection of manufacturing cell lines by other criteria (growth rate, response to feed, genetic stability, etc.) and also enables the isolation of high producers early in the cell line engineering process. Thus, more effort can be directed to characterize clones of high productivity.

**Figure 5:**
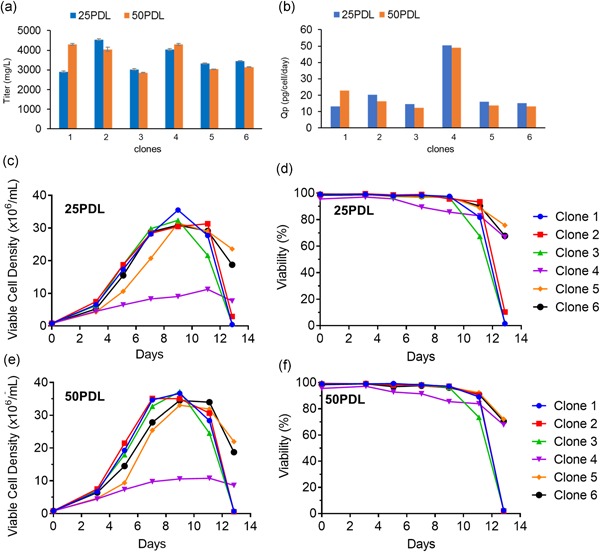
Clones isolated by surface display show phenotypic stability over 50 generations. The six highest IgG‐expressing clones identified were assessed for long term stability in duplicate (*n* = 2). (a) Expression titers and their standard deviations were determined for the selected clones (1–6) by 13‐day fed‐batch culture after 25 and 50 generations of growth. (b) Specific IgG productivity (Qp or pg/cell/day) was calculated for each expressor cell line. All clones were derived from the high surface‐display pool except clone 4, which was identified in the nonenriched group. All clones showed stable harvest titer expression levels and specific productivity. Viable cell densities and viabilities are shown for 25PDL (c, d) and 50PDL (e,f) cells in fed‐batch. The overall kinetics are similar between the two generations. IgG: immunoglobulin G; PDL: population doubling level [Color figure can be viewed at wileyonlinelibrary.com]

Although it is unlikely that the process of surface‐display based selection would introduce genetic instability into the expressor population beyond what is normally observed, we examined the phenotypic stability of the six highest expressing clones for antibody production. Clones, selected from the high surface‐display gate (*n* = 5; clones 1,2,3,5, and 6) and nonenriched population (*n* = 1; clone 4) were propagated for 50 generations and antibody titers and specific productivity were assessed. All six clones demonstrated consistent intracellular antibody expression (Supporting Information Figure S4), secreted antibody productivity (Figure 5a,b), and culture kinetics (Figure 5c–f) irrespective of passage number over a 13‐day fed‐batch expression. Expression levels in all clones were lower in fed‐batch shake flasks than what was observed in the high‐throughput screen performed in 96 deep‐well plates, where evaporation of the medium can result in an apparent increase in productivity. The expressed antibodies from each of these cell lines were also assayed to detect the presence IgG containing the GPI anchor. Although pylRS/tRNApyl previously has been shown to have no amber suppression activity in the absence of nnAA, purified IgG from the fed‐batch cultures (without nnAA) of the same six top expresser clones was examined by mass spectrometry. The results show monoisotopic peaks corresponding to the molecular mass of IgG HC (theoretical mass, 51.27 kDa) and LC (23.45 kDa), but no signal corresponding to the GPI‐anchored heavy chains (53.73 kDa; Supporting Information Figure S5). These data suggest that the vast majority of the IgG does not contain the GPI anchor, ensuring the homogeneity of the product.

### 3.4 Clonal enrichment of difficult‐to‐express proteins

**Figure 6:**
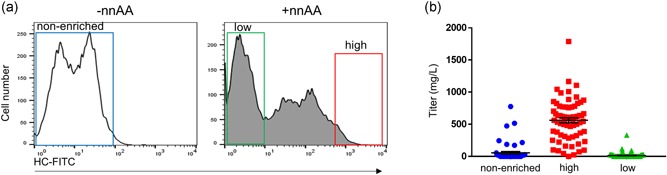
Surface display enhances the identification of high expressors for a difficult‐to‐express molecule. (a) Cells stably expressing a difficult‐to‐express molecule (MEDI‐X) were subjected to surface display and high and low gates used to select single cells. Clones derived from a nonenriched population were also isolated. (b) Isolated clones were assessed for expression titer in 96 deep‐well fed‐batch culture after Day 14. Clones derived from a high surface‐display gate show not only higher overall expression titer levels, but also higher numbers of high‐producing clones [Color figure can be viewed at wileyonlinelibrary.com]

Complex recombinant molecules are emerging as the next generation of therapeutics. These highly engineered proteins include bispecific antibodies (e.g., BiTES, DARTS, and IgG‐scFvs) and fusion proteins that represent important new medicines with enhanced functionality for disease treatment (Spiess, Zhai, & Carter, 2015). However, these molecules are often labeled as “difficult to express” due to the low expression titer and low specific productivity (Kontermann & Brinkmann, 2015). Thus, manufacturers face difficulties in producing these proteins. To address the bottleneck, we investigated the use of the surface‐display method to isolate high‐producer cells for a difficult‐to‐express target. To do this, CHO‐RS cells were stably transfected with a plasmid encoding a proprietary bispecific antibody (MEDI‐X) bearing the reversible GPI‐membrane anchor. MEDI‐X is a symmetrically bispecific antibody consisting of an IgG with an inserted scFv in the CH3 domain (Cao et al., 2018). This bispecific was selected in part as an extensive conventional screen had been recently conducted that resulted in the isolation of cell lines capable of 1 g/L yields. We thus subjected the transfected cells to surface display and selection from both high and low surface‐staining gates to determine whether this method could improve on the previously observed yields (Figure 6a). A control population sorted without surface display was generated (nonenriched) in parallel. The recovered clones were expanded and their productivity was determined in 96 deep‐well plates by the fed‐batch culture at Day 14 (Figure 6b). The top clone from the nonenriched population achieved a titer of ~800 mg/L that is consistent with previous efforts. However, with surface display we saw, not only a significant improvement in the titers of the top expressor (up to 1.8 g/L), but also 18 additional clones with titers above 800 mg/L, including five clones with yields exceeding 1 g/L. These data illustrate the potential of this technology for the enrichment of high‐expressor clones.

## 4 DISCUSSION

Isolating high‐producing cell lines is key for the biotherapeutic protein drug development process. There has been a substantial improvement in the yield of CHO cell‐based production processes in recent years, and yields exceeding 10 g/L have been reported (Kunert & Reinhart, 2016). However, clones having this high production potential are extremely rare in populations of transfectants generated by random integration of expression plasmids, so significant screening efforts are required to identify and isolate them. In an effort to consistently generate high‐producing clones, with better genetic stability and reduce the screening effort, site‐specific integration methods targeting transcriptionally active genetic hotspots have been implemented (Lai, Yang, & Ng, 2006). This method has been reported to be very successful and shown to reproducibly reach high g/L titers. But the most successful loci are closely guarded secrets and unavailable for common use. Thus, random integration of expression genes remains a stalwart for cell line engineering. In this study, we describe a simple and feasible method to rapidly isolate highly productive cells for the biomanufacturing process using reversible anchoring and surface display of the therapeutic protein at the expressing cell membrane. The key feature of this approach is the ability to combine amber suppression‐induced surface binding with the enrichment or cloning step, thereby isolating cells with better productivity. The selection of cells is performed in a single step early in the cell line engineering process, greatly reducing the screen size, and using FACS instrumentation that is already in use in many facilities. Our results demonstrate that levels of cell surface display of antibodies in CHO cells correlates with their productivity, allowing us to accurately eliminate low and nonproducer populations at an early stage of the FACS cloning process and leading to efficient enrichment for high‐producing clones.

Previous efforts to identify rare, high‐producing cells have largely focused on noncovalent immobilization/interaction of secreted proteins on the cell surface (Cost et al., 2010; Dreesen & Fussenegger, 2011; Mazur, Fussenegger, Renner, & Bailey, 1998; Roy, Miro‐Quesada, et al., 2017; Zhou et al., 2011). The main advantage of these is that expressing cells are phenotypically selected for both expression and secretion, two key properties of the best producers. However, cell surface immobilization approaches are lengthy processes, often resulting in low cell viability. Moreover, secreted proteins that are not immobilized on the surface of the expressing cells can diffuse into the medium and be bound nonspecifically to the surfaces of nearby nonproducer cells, which can then be selected in the FACS step, increasing the background, and making the method less predictive of product yield. There are other methods that involve detection of antibody expression on the cell surface via a transient anchoring strategy or using surrogate genes (Bailey et al., 2002; DeMaria et al., 2007; Helman et al., 2014; Kumar & Borth, 2012; S. Lang et al., 2016); however, our method possesses the advantage of “switchable” amber suppression in response to the addition or removal of the nnAA in the medium that enables a product‐dependent isolation method. Our FACS‐based transient surface‐display strategy discriminates and segregates high‐, low‐ and nonproducing cells during sorting. We have shown that stably transfected cells displaying high nnAA‐induced surface binding have enhanced productivity relative to the untreated cells. Moreover, by sorting for high surface display, we were able to eliminate clones with undesirable productivity at an early stage of the CLD process and consequently achieved efficient enrichment of high‐producer clones from heterogenous stable transfectant pools generated by random integration. Furthermore, we examined different IgG molecule formats with varying levels of expression, namely an IgG mAb, and a difficult‐to‐express bispecific antibody (MEDI‐X). In both cases, selection using surface display resulted in clones with improved expression titers than control screens. With a conventional antibody, the overall titers obtained using surface display (>8.5 g/L) were similar to those of the control screen. However, the rate of identification of these clones was improved by the surface display (7.8% or 6 clones) than controls (1.4% or 1 clone). Improved overall titers were observed with difficult‐to‐express proteins such as MEDI‐X. Here, surface display resulted in a greater than two‐fold improvement in yields over the control process (1.8 g/L vs 800 mg/L) as well as an enrichment in high producers. It is possible that with conventional antibodies the expression rates are near maximum with little room for greater productivity. This may not be the case with MEDI‐X, where improvements in productivity are below what is physiologically achievable by the cells.

Product quality and stability are as critical as productivity in the CLD process. A longstanding problem in CLD has been heterogeneity of expression levels of cells within a clone contributing to the instability of the expression level and potentially causing changes in product quality attributes over time (Ko et al., 2017; Pilbrough, Munro, & Gray, 2009). Phenotypic variation may develop as early as 18 days after cloning and the heterogeneity in expression among the cells of a clone can rapidly become comparable to that of mixed transfectant pools. In our study, the top clones generated from a phenotypically diverse cell population based on high surface display, demonstrated consistent antibody productivity over the course of 50 generations indicating a high degree of gene expression stability. In addition to expression stability, other criteria including cell growth and doubling time, achievable cell densities, glycosylation, and viability at harvest are evaluated in the final selection of the manufacturing cell line. By virtue of enriching for high producers, a greater number of cell lines are available for selection by these other criteria that may enhance the overall manufacturability of the protein product.

The surface display method described here exploits a physical link between the expressing cell and it’s recombinant protein product that allows for the direct identification of high‐producing cells. This is enabled by the inducible nature of the surface display method, which unlike analogous technologies relying on leaky stop codons, allows a regulated start for the surface display (S. Lang et al., 2016). The increased level of control allows for optimization of the conditions for cell selection. Indeed, we observed that extended periods of induction led to a saturation of the cell membrane resulting in a reduced ability to discriminate high‐producing clones from lower expressors. The switchable nature of the system also enables a rapid transition between the surface display and product generation without additional cloning. The highly regulated orthogonal pylRS/tRNApyl has been shown to efficiently incorporate nnAA at amber codons in the presence of nnAA, but with no detectable readthrough in its absence eliminating the purification of GPI‐anchored proteins (VanBrunt et al., 2015). Another advantage of this system over methods relying on intracellular surrogate staining, is that selection requires both high expression and secretion of a molecule that is highly related to the product. The regulation of surface display requires the use of engineered host cell lines with amber suppression capabilities, so while this technology may not be widely available, several orthogonal tRNA synthetase/tRNAs have been developed for nnAA incorporation in mammalian cells that could be readily adapted for this purpose (Italia et al., 2017; Mukai et al., 2008; Schmied, Elsässer, Uttamapinant, & Chin, 2014). Furthermore, the system is not restricted to identifying CHO cells expressing high levels of antibodies and may be readily applied to a variety of protein classes including other secreted proteins (both antibody and nonantibody) as well as transmembrane proteins (Britton et al., in‐press). Taken together, our present study demonstrates a widely applicable FACS gating strategy based on nnAA‐induced surface display for isolation of phenotypically stable high‐producing cells. We believe that this technique should provide a rapid, efficient, and high‐throughput tool for the CLD process for production of recombinant proteins.

## CONFLICTS OF INTEREST

All authors are employees and shareholders of MedImmune/AstraZeneca.

## Supporting information

Supporting information

Supporting information

Supporting information

Supporting information

Supporting information

Supporting information

Supporting information
