# Supplementary figures and images for: Amber suppression coupled with inducible surface display identifies cells with high recombinant protein productivity
Source: Biotechnol Bioeng. 2019 Jan 18;116(4):793–804. doi: 10.1002/bit.26892 (PMC6590230; doi:10.1002/bit.26892)

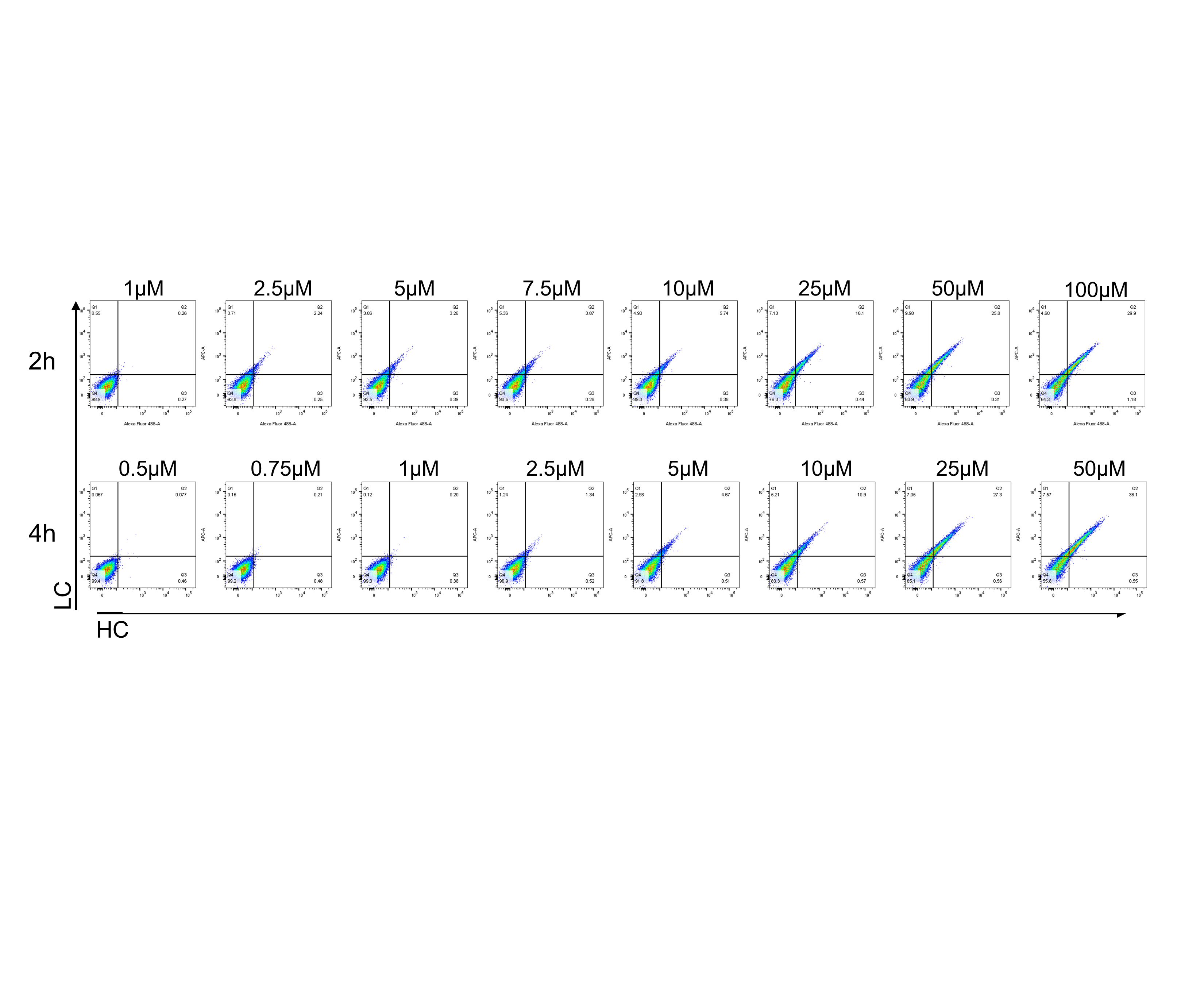

Supplement: Supplementary file 1 — Supporting information [file BIT-116-793-s001.TIF]

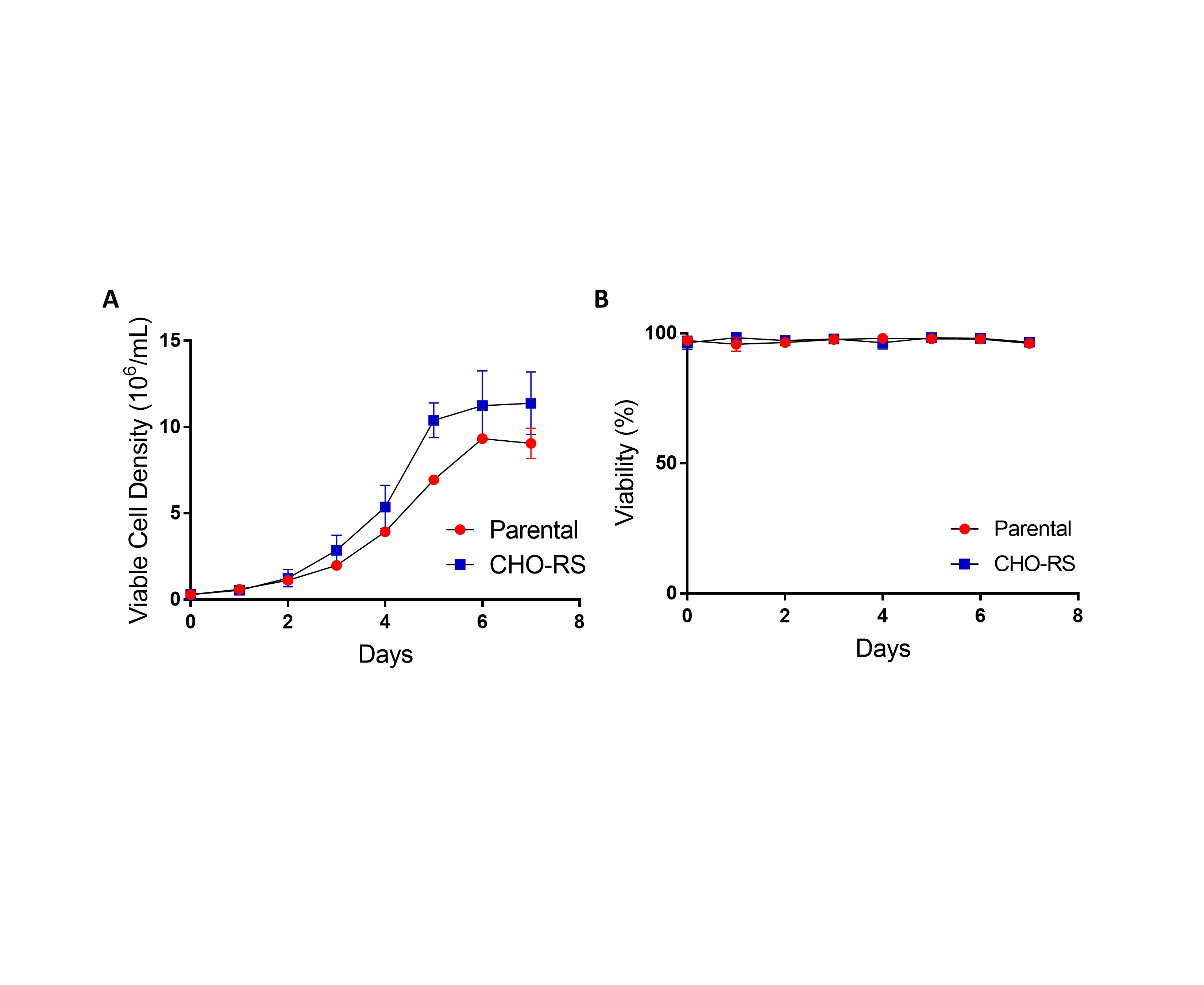

Supplement: Supplementary file 2 — Supporting information [file BIT-116-793-s002.TIF]

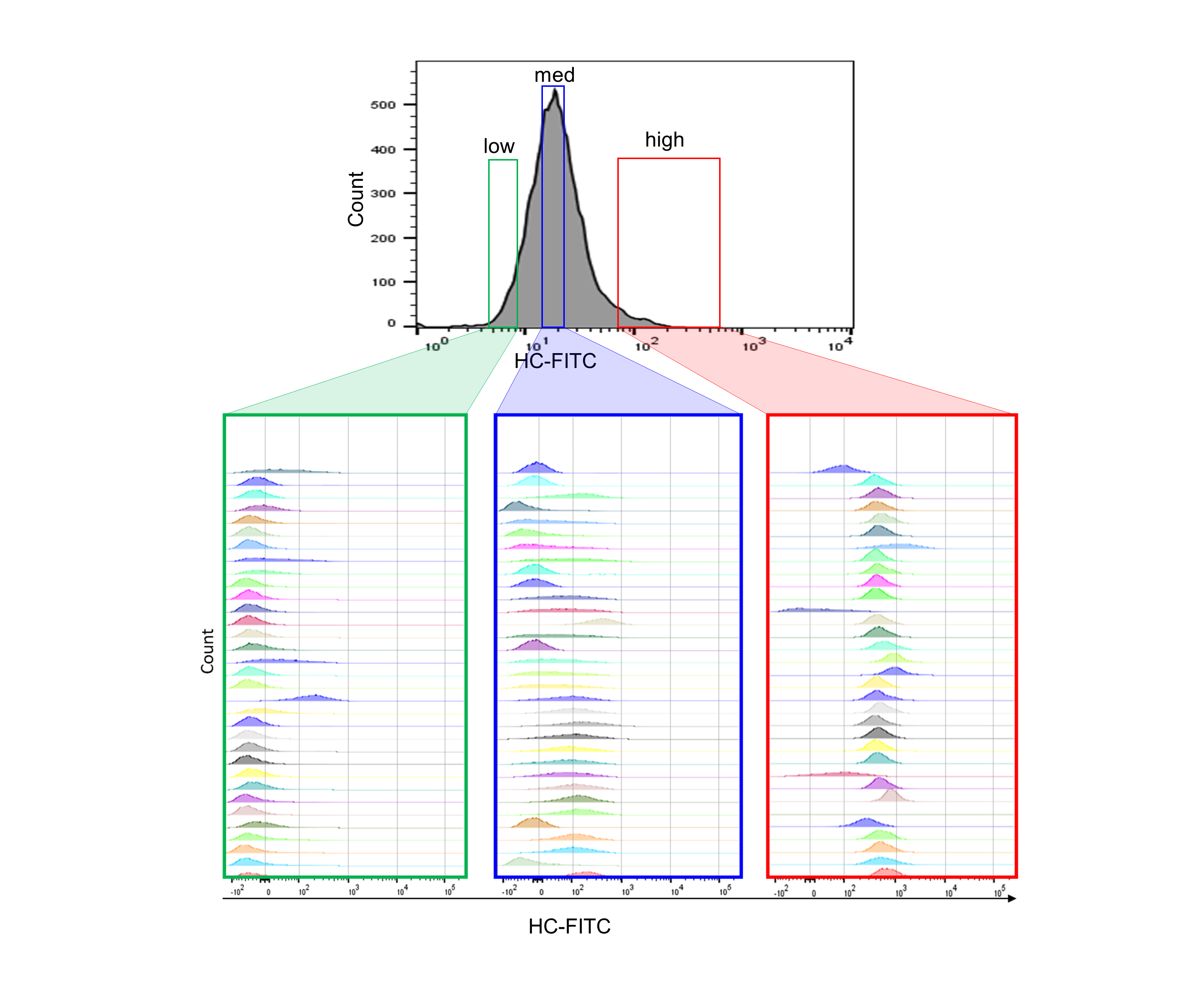

Supplement: Supplementary file 3 — Supporting information [file BIT-116-793-s003.TIF]

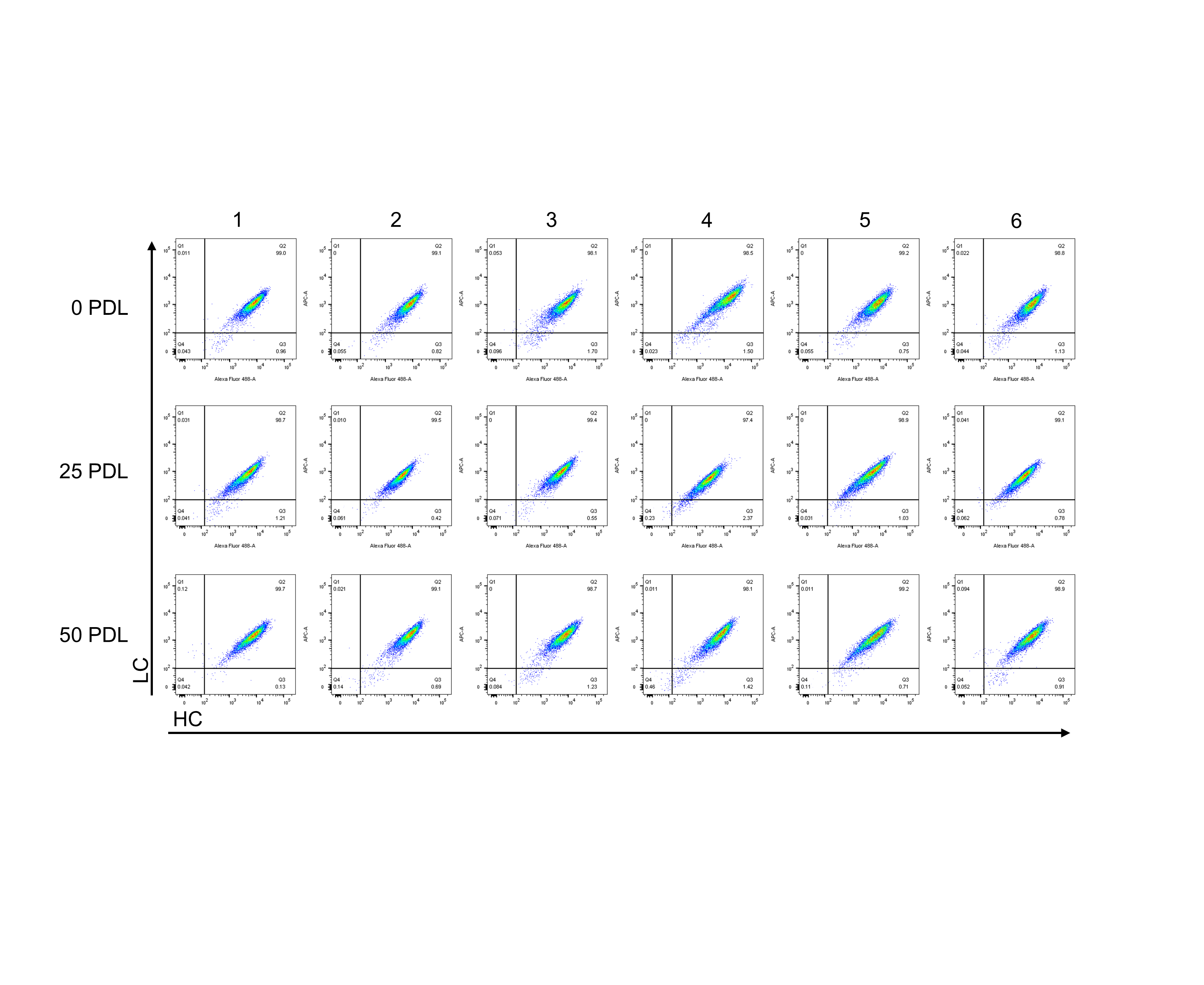

Supplement: Supplementary file 4 — Supporting information [file BIT-116-793-s004.TIF]

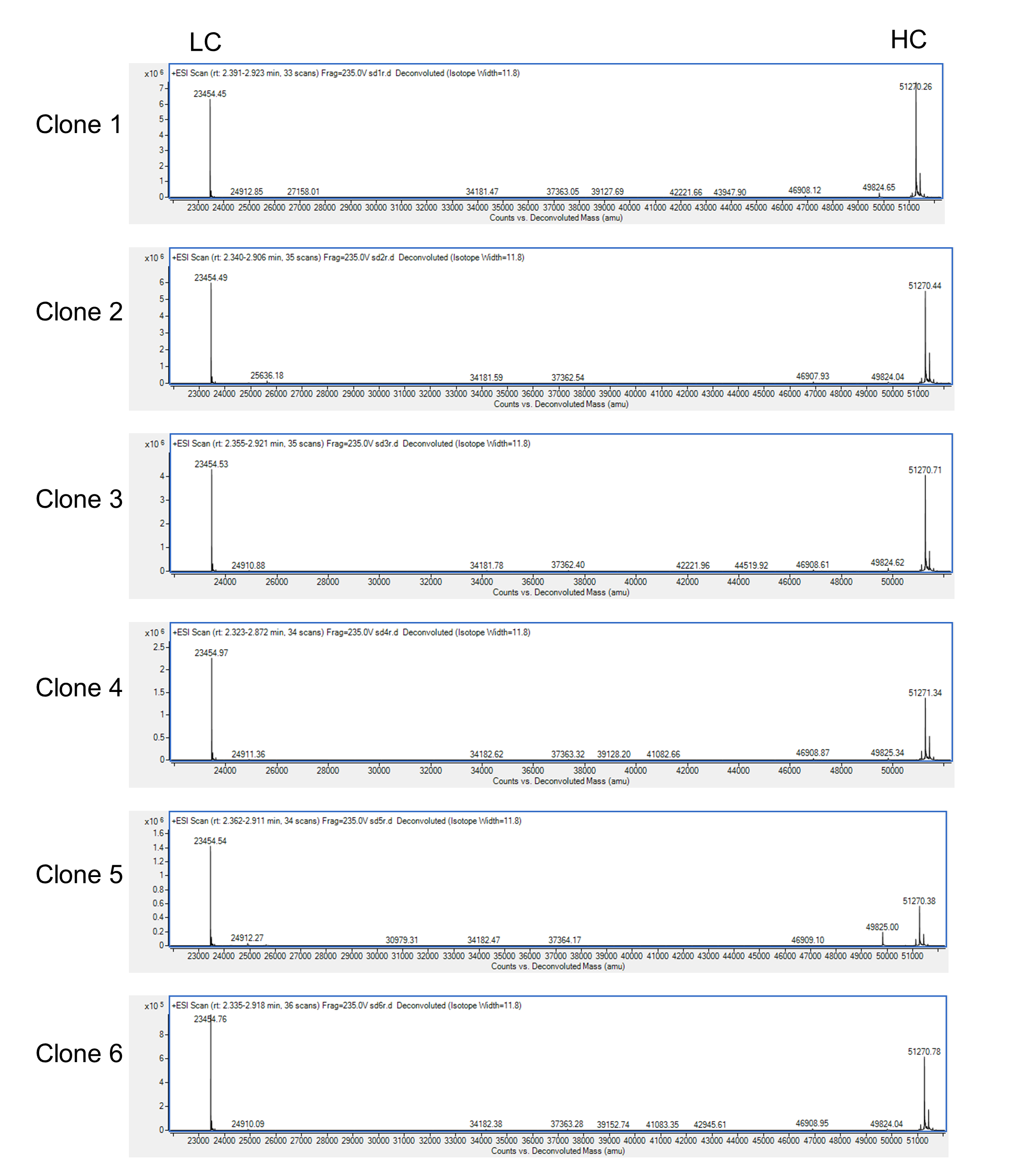

Supplement: Supplementary file 5 — Supporting information [file BIT-116-793-s005.tif]
