# Supplementary material for: Amber suppression coupled with inducible surface display identifies cells with high recombinant protein productivity
Source: Biotechnol Bioeng. 2019 Jan 18;116(4):793–804. doi: 10.1002/bit.26892 (PMC6590230; doi:10.1002/bit.26892)
Supplement: Supplementary file 6 — Supporting information [file BIT-116-793-s006.docx]

## Supplementary Figure Legends

**Supplementary Figure S1. Surface display of IgG-GPI is tunable**. CHO-RS cells stably transfected with IgG-GPI-Amber were grown in the indicated concentrations of nnAA for 2 or 4 h. Cells were then stained with fluorescently labeled antibodies specific for the HC and LC of the IgG and the population analyzed by flow cytometry. The stained population increased concomitant with nnAA concentration and time. For this highly expressed molecule, 25µM nnAA and 2h of incubation was selected as an optimal condition that enables differentiation of cells based on surface staining while maintaining non-saturating staining levels.

**Supplementary Figure S2. Growth properties of parental and engineered host cells.** Growth properties of the parental and engineered host were measured in shake flask cultures. Parental and engineered hosts (CHO-RS) were seeded at 300,000 cells per mL in CD-CHO medium and grown at 37°C for 7 days. Viable cell density (**A**) and overall viability (**B**) were measured daily and the average and standard deviations of two experiments are shown. CHO-RS shows similar growth kinetics and viability to the parental cell line, indicating that the PylRS and tRNA expression does not affect their overall growth.

**Supplementary Figure S3**. **HC MFI curves for clones selected from low, medium and high surface display gates**. 33 clones expressing IgG selected from high, medium and low surface display were expanded and HC surface display levels retested. Clones selected from the high gates retain elevated IgG display properties relative to low and medium gated clones. Most of the high expressing clones show a narrow distribution of expression levels indicating high homogeneity within each population.

**Supplementary Figure S4**. **Stability of IgG expression was assessed in selected clones by intracellular staining**. The six highest expressing clones isolated by either surface display or from a non-enriched population (clone 4) were passaged for 50 generations to assess changes in IgG expression. Intracellular IgG was quantified by flow cytometry after 0, 25 and 50 generations. No observable changes were seen in any of the tested clones suggesting stable IgG expression over 50 generations.

**Supplementary Figure S5**. **Mass spectrometric analysis of secreted IgG**. IgG from each of the six highest expressing clones isolated by either surface display or from a non-enriched population (clone 4) were purified by affinity chromatography and reduced samples analysed by mass spectrometry. All clones showed signals with molecular mass corresponding to LC (23.45KDa), and glycosylated HC (51.27KDa). No peaks corresponding to the HC-GPI (53.73+ KDa) were observed.
