# Supplementary material for: Amber suppression coupled with inducible surface display identifies cells with high recombinant protein productivity
Source: Biotechnol Bioeng. 2019 Jan 18;116(4):793–804. doi: 10.1002/bit.26892 (PMC6590230; doi:10.1002/bit.26892)
Supplement: Supplementary file 7 — Supporting information [file BIT-116-793-s007.docx]

**Supplementary Table 1. Surface display results in the enrichment of high expressors.**

| Gate | Percent of cells | | | | |
| --- | --- | --- | --- | --- | --- |
| **Expression titer (g/l)** | **<5** | **>5** | **>7** | **>8.5** | **N** |
| **non-enriched** | 43.1 | 33.3 | 22.2 | 1.4 | 72 |
| **High** | 16.9 | 53.2 | 22.1 | 7.8 | 77 |
| **Med** | 58 | 34 | 8 | 0 | 50 |
| **Low** | 70.4 | 29.6 | 0 | 0 | 81 |

Titers from clones derived from non-enriched, high medium and low gates of surface display were examined. The percentage of cells displaying titers >8.5, >7, >5 and <5g/L were calculated. High surface display cells show an enrichment of cell lines with productivity above 8.5g/L (7.8%) and selective exclusion of low producers (<5g/L; 16.9%). In contrast, non-enriched cells show 1.4% of high and 43% of low producers. N; Number of clones analyzed in each group.
